# Surgical strategy for intravenous leiomyomatosis spreading from uterine to the right atrium presenting with recurrent syncope

**DOI:** 10.1186/s13019-024-02681-3

**Published:** 2024-04-15

**Authors:** Gengxu He, Tong Yao, Lei Zhao, Hong Geng, Qiang Ji, Kun Zuo, Yuanzhi Luo, Kai Zhou

**Affiliations:** 1 Department of Thoracic and Cardiovascular Surgery, The First Affiliated Hospital of Hebei North University, Zhangjiakou City, Hebei Province P. R. China; 2 Department of Cardiac Function, The First Affiliated Hospital of Hebei North University, Zhangjiakou City, Hebei Province P. R. China

**Keywords:** Intravenous leiomyomatosis, Cardiopulmonary bypass

## Abstract

Uterine leiomyoma invading internal iliac vein and consequently disseminating into the right atrium is an extremely rare condition, and surgical strategy is controversial. Here, we reported a specific case with successful surgical resection through one-stage total hysterectomy, bilateral oophorectomy, and the intracardiovascular lesion. This procedure would be an optimal choice for uterine leiomyoma invading inferior vena cava and spreading to right atrium.

## Background

Although uterine leiomyoma is common among premenopausal women, intravenous leiomyomatosis is a relatively rare condition, accounting for 10% of patients [1, 2]. Intravenous leiomyomatosis is characterized by proliferation of neoplastic smooth muscle cells within venous drainage system of abdomen and pelvis, often secondary to direct intravascular extension of original uterine leiomyomata. For its wide invasion, the surgical strategy is controversial, In this case report, one stage surgical resection of intravascular leiomyomatosis was performed in a patient presenting with intermittent syncope as leiomyoma floating in the right atrium and intermittently obstructing tricuspid valve.

### Clinical data

**Fig. 1:**
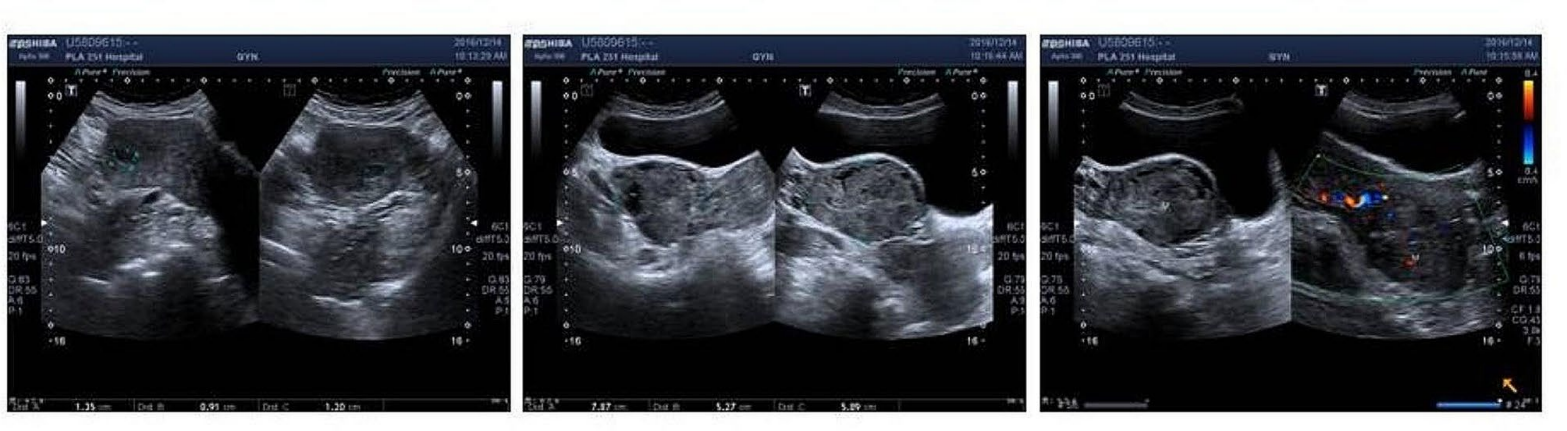
Pelvic ultrasonography identified obviously enlarged uterine mass, with a diameter of 13 cm

**Fig. 2:**
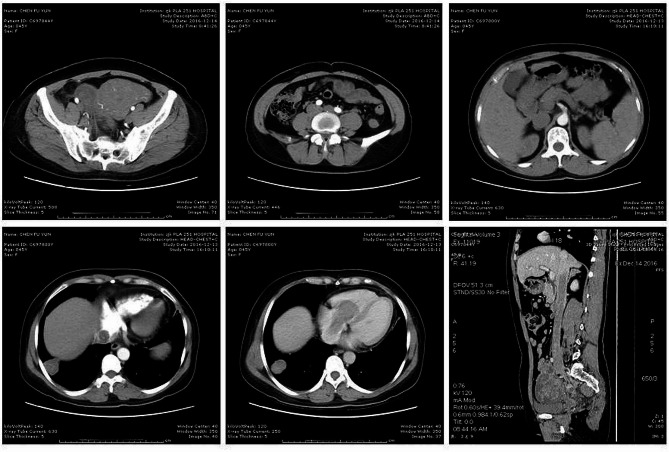
Computed tomography (CT) of pelvic cavity and heart identified a 13.7 × 5.4 cm [2] sized, irregular shaped, unclear boundary, and soft tissue density mass within the uterus. The tumor extended through inferior vena cava into the right atrium and ventricle

**Fig. 3:**
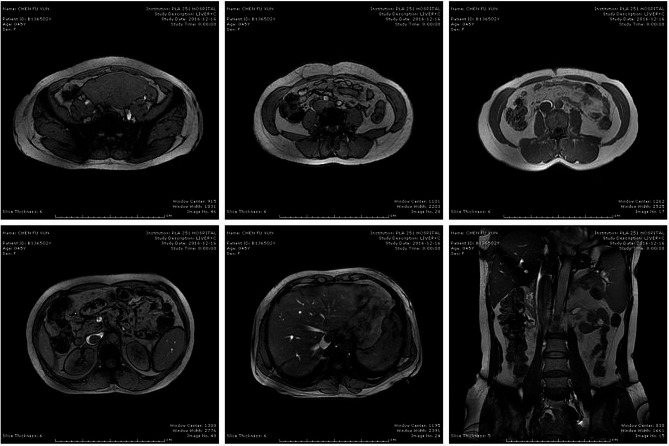
Enhanced Magnetic Resonance Image of abdomen revealed obviously enlarged uterine and bilateral ovaries, with no clear boundary. The tumor extended through venae iliaca interna to inferior vena cava

A 45-years-old woman, presenting with shortness of breath for 6 months, and intermittent syncope for 8 times, consulted a nearby clinic. The echocardiography identified a solid tumor with the maximum diameter of 7.5 cm in the right atrium, floating into the right ventricle during cardiac diastole. This patient was transferred to Department of Thoracic and Cardiovascular Surgery, The First Affiliated Hospital of Hebei North University. Immediately, a serial examination was carried out. Pelvic ultrasonography revealed an obviously enlarged uterine mass, with a diameter of 13 cm (Fig. 1). Computed tomography (CT) of pelvic cavity identified a 13.7 × 5.4 cm [2] sized, irregular shaped, unclear boundary, and soft tissue density mass within her uterus (Fig. 2). Contrast-enhanced CT angiography suggested a strip of filling defect in the right ventricle and the right atrium, extending from inferior vena cava. Multiple round-shaped nodules of different sizes were randomly distributed in bilateral lung field (Fig. 2). Enhanced magnetic resonance image (MRI) of abdomen revealed obviously enlarged uterine and bilateral ovaries, loss of clear boundary, and a tumor extending through venae iliaca interna to inferior vena cava (Fig. 3). This patient was diagnosed with intravenous leiomyomatosis originating from uterine leiomyomata, and with pulmonary metastatic leiomyoma.

### Surgical procedure

**Fig. 4:**
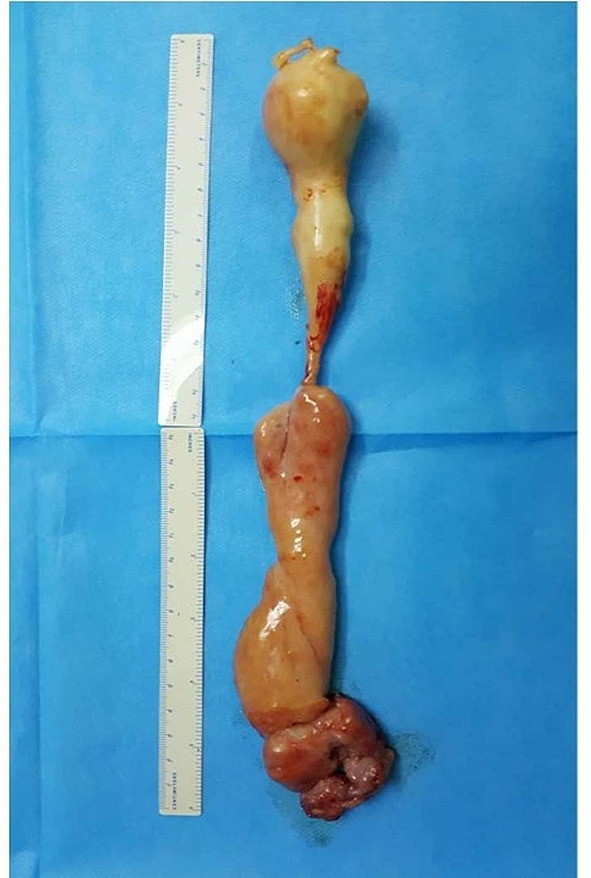
The surgical specimen removed from inferior vena cava and the right atrium

After routine examination, we carried out one-stage total hysterectomy, bilateral oophorectomy, and removal of intracardiovascular lesions. After anesthesia, this patient was placed in supine position on the operating table. First, the cardiac surgeon opened the patient’s chest through the median sternotomy, and cut the pericardium to expose the heart. Then gynecologist carried out total hysterectomy and bilateral salpingo-oophorectomy through the midline incision in the lower abdomen. The uterine and bilateral ovaries were resected smoothly and the right internal iliac vein was exposed. The tumor located in the right internal iliac vein had spread to the right common iliac vein. The right internal iliac vein was opened and the tumor was totally resected. After systemic heparization, the cardiac surgeon established cardiopulmonary bypass (CPB) as follows. An aorta cannulation was inserted into ascending aorta, and two drainage- cannulas were inserted into superior vena cava and the right atrium, respectively. Then, CPB was started. During parallel circulation, superior vena was occluded; the right atrium was opened through an oblique incision; a part of tumor in the right atrium was exposed and gently pulled from the inferior vena cava. Afterwards, the cannula in the right atrium was inserted into inferior vena cava, the rest of the tumor was completely removed (Fig. 4). The right atrial incision was closed. After rewarming to normal temperature, CPB was stopped and the cannulas were removed one by one. The whole procedure lasted about 6 h. The patient rehabilitated completely.

According to postoperative histological examination, tumors presented as spindle-like smooth muscle cells without atypia, accompanied with local mucus and hyaline degeneration. Immunohistochemical stain exhibited desmin (+), smooth muscle actin (SMA) (+), S-100 (-), estrogen receptor (ER) (+), and progesterone receptor (PR) (+). The patient was followed up for 5 years. There are no signs of recurrence in pelvic cavity, nor progression of pulmonary nodules.

## Discussion

Intracardiac extension complicated with metastatic pulmonary leiomyoma belongs to a rare condition of intravenous leiomyomatosis. Obstruction of tricuspid valve or the right ventricle outflow tract can be always fatal, while surgical resection remains the only therapeutic approach.

Involvement of intravenous leiomyomatosis (IVL) is very rare, while surgical strategy for treatment is challenging. Intravenous leiomyomatosis can be categorized into four clinical stages according to tumor progression to different levels of inferior vena cava system [3] Recent study has recommended that one-stage surgery could be an optimal choice, except when a patient’s general condition is poor, or a patient has a huge vascular tumor, serious abdominal and pelvic adhesion [3]. As for stage III and IV diseases, tumor invades renal vein or inferior vena cava, or more proximally into the right atrium or pulmonary artery, one-stage removal of tumor may be an optimal choice [4]. In our case, we evaluated tumor stage preoperatively using contrast-enhanced CT and enhanced MRI. This tumor did not adhere to the right common iliac vein and inferior vena cava, and had a smooth surface. Accordingly, one- stage surgery was carried out. During surgical procedure, the first step was to open the chest through median sternotomy to prepare for cardiopulmonary bypass. The tumor floats into tricuspid valve and obstructs the right ventricle outflow tract. The second step was to perform total primary tumor resection, hysterectomy, bilateral salpingo-oophorectomy and removal of original part of metastatic tumor in the right internal iliac vein. The third step was to establish cardiopulmonary bypass and pull metastatic tumor gently from the right atrium and inferior vena cava. Intraoperative observation revealed a smooth surface of IVL, with endothelial coverage and no thrombosis. The texture of IVL is solid, and easy to remove, which rarely causes embolisms. There was no adherence between IVL tumor and vascular system. The tumor was easily pulled out from the right ventricle and inferior vena cava intactly.

Currently, pathological mechanisms of IVL remain unclear. Possibly, IVL may be originated from vascular wall [5] or when uterine leiomyoma invading blood vessels [6]. Additionally, IVL can follow via two distinct routes into systemic venous circulation: uterine vein (majority) and ovarian vein (minority) [7]. IVL stemmed from uterine vein can extend into internal iliac veins, common iliac veins, inferior vena cava and even the right atrium.

There are several reports about pulmonary metastasis of leiomyoma simultaneously with intravascular lesions [8–14]. Based on histopathological analysis, metastatic lesions and IVL were closely related to each other [15]. Pathogenesis of benign metastasizing leiomyoma (BML) remains mysterious [8, 16]. In our case, BML involving the lung was asymptomatic, which could be monitored at follow-up, requiring no immediate treatment. During 5-years’ follow up, pulmonary nodules remained stable, without sings of malignant transformation. In the absence of total resection of hysterectomy, bilateral salpingo-oophorectomy, long-term treatment with gonadotropin-releasing hormone agonists may be useful in preventing recurrence of this disease [17, 18].

In conclusion, one-stage resection including IVL extending into the right atrium, uterus, and bilateral salpingo and oophoron will be an optimal option for IVL extending from uterine to the right atrium.

## Data Availability

The datasets used and/or analyzed during the current study are available from the corresponding author on reasonable request.
